# US Women's Perceptions and Acceptance of New Reproductive Health Technologies

**DOI:** 10.1089/whr.2020.0063

**Published:** 2020-09-24

**Authors:** Amber K. Worthington, Erin E. Burke, Talia N. Shirazi, Carly Leahy

**Affiliations:** ^1^ Department of Communication, University of Alaska Anchorage, Anchorage, Alaska, USA.; ^2^ Modern Fertility, San Francisco, California, USA.

**Keywords:** fertility testing, online consultation, self-collection test, technology acceptance, telemedicine, reproductive hormones

## Abstract

***Background:*** Women have faced persistent problems accessing reproductive health care. New applications of health technologies to reproductive health, specifically online fertility specialist consultations and reproductive hormone self-collection tests (SCTs), present unique opportunities to overcome these issues. This article uses the technology acceptance model to examine factors that influence women's intentions to use these new reproductive health technologies.

***Materials and Methods:*** Participants (*n* = 327 US women) completed an online survey assessing perceptions related to both of these reproductive health technologies, including usefulness, ease of use, risk, trust, subjective norms, and personal responsibility, to learn about fertility.

***Results:*** Participants indicated high perceptions of usefulness, ease of use, and trust, as well as low perceptions of risk and subjective norms for both online fertility consultations (OFCs) and reproductive hormone SCTs. Women indicated low perceptions of responsibility to use OFCs, but high perceptions of responsibility to use reproductive hormone SCTs. Structural equation modeling indicated that intentions to use OFCs were predicted by usefulness, subjective norms, and responsibility; intentions to use reproductive hormone SCTs were predicted by usefulness, ease of use, subjective norms, and responsibility.

***Conclusions:*** Fertility specialist consultations and reproductive hormone testing can provide women with essential fertility information that facilitates informed reproductive decisions; however, these services have historically been difficult to access. Widespread uptake of new reproductive health technologies could promote positive advances in women's reproductive health outcomes.

## Introduction

Recent trends show that women are delaying childbirth, and total fertility rates are decreasing.^1,2^ Women's access to reproductive health care services, including fertility specialists and reproductive hormone testing, is thus increasingly important. Indeed, fertility specialists have a greater understanding of fertility and can counsel women about natural age-related declines in fertility to help them realize their reproductive goals.^3^ Furthermore, reproductive hormone tests provide women with important information about their hormone levels, ovarian reserve, and fertility window^4^ and, again, can facilitate women's ability to achieve their reproductive goals. Past work has found that the information from reproductive hormone tests could lead women to alter their plans by, for example, trying to conceive children earlier or using fertility preservation technologies.^5–7^

Importantly, persistent problems have plagued women's access to these reproductive health care services. Many women face difficulties seeking care from a fertility specialist due to provider scarcity, geography, time, and cost.^8,9^ In the United States, there were only 463 fertility clinics reporting to the Centers for Disease Control and Prevention in 2016.^10^ Furthermore, even if women have access to a fertility specialist, infertility is a stigmatized condition,^11,12^ and women may feel uncomfortable seeking fertility health care from a specialist as people often delay or avoid seeking treatment for stigmatized conditions.^13,14^ Women have also faced difficulties accessing reproductive hormone testing due to cost,^15,16^ and women often lack the reproductive health education necessary to understand the importance of and results from these tests.^17^

**FIG. 1.**
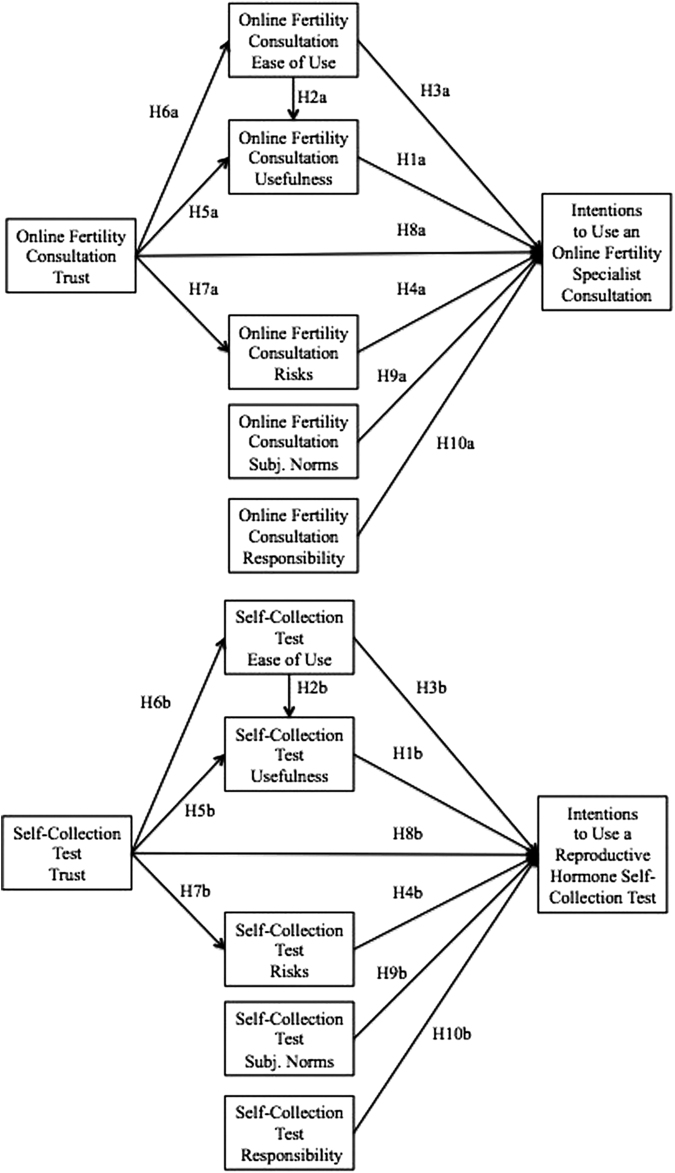
Theoretical models predicting intentions to use an online fertility specialist consultation and reproductive hormone SCT. SCT, self-collection test.

New applications of health technologies have created an opportunity to overcome these barriers in accessing reproductive health care. Across sexual health care, there have been rapid increases in telemedicine^18,19^ and self-collection tests (SCTs)^20,21^ that have mitigated barriers to access, while still maintaining a high standard of patient treatment and satisfaction.^18,22,23^ Within reproductive health care, it has recently become possible for women to seek care from fertility specialists through online fertility consultations (OFCs) and for women to use low-cost, reproductive hormone SCTs.^4^ As mentioned, the use of these tools can improve women's reproductive health outcomes. Thus, it is important to investigate factors that influence women to accept and use OFCs and reproductive hormone SCTs. The technology acceptance model (TAM)^24,25^ and extensions, including perceived risk and trust,^26^ subjective norms,^27^ and responsibility,^28^ are useful for understanding new technology acceptance. The goal of this study was thus to examine factors that influence women's acceptance of these new reproductive health technologies using the TAM. Figure 1 displays our theoretical models.

### TAM: perceived usefulness and perceived ease of use

The TAM^24,25^ examines factors that influence an individual's intended use of a particular technology. The TAM posits that intentions to accept and use a technology are directly influenced by perceived usefulness and perceived ease of use, and perceived usefulness is predicted by perceived ease of use. Perceived usefulness is defined as the degree to which an individual believes that using a particular technology would be beneficial, and both OFCs and reproductive hormone SCTs may be perceived as useful. Indeed, telemedicine allows health care providers to offer the same services they provide to patients during in-person consultations,^18,19^ and studies directly comparing phone- or video-based consultations to in-person health care consultations suggest similar patient outcomes and satisfaction.^22,23^ Together, this suggests that online consultations may be as useful as traditional in-person health care consultations. SCTs can be used interchangeably with venipuncture sampling to measure reproductive hormones,^4^ and a small-scale ethnographic study found that their participants believed the results from reproductive hormone SCTs were empowering.^29^ Again, this suggests that these reproductive SCTs may also be as useful as traditional reproductive hormone testing in in-person or laboratory settings.

Perceived ease of use is defined as the degree to which an individual believes that using a particular technology would be free from effort, and both OFCs and reproductive hormone SCTs may also be perceived as easy to use. Indeed, past research has found that both online consultations^18,30^ and SCTs^21^ for a wide range of health services circumvent the geographic, financial, and time-based limitations of in-person health care services.^8,31^ Both OFCs and reproductive hormone SCTs can also be used discreetly and in the privacy of one's own home, thus potentially mitigating some of the stigma felt by women pursuing fertility care.^12^ Both OFCs^32^ and reproductive hormone SCTs^4^ are commercially available; however, to date, there are no published studies of women's perceived usefulness and ease of use of these reproductive health technologies. In line with the TAM, we hypothesized the following:

H1: Perceived usefulness is positively related to intentions to use (a) online fertility consultations and (b) reproductive hormone self-collection tests.

H2: Perceived ease of use is positively related to perceived usefulness of (a) online fertility consultations and (b) reproductive hormone self-collection tests.

H3: Perceived ease of use is positively related to intentions to use (a) online fertility consultations and (b) reproductive hormone self-collection tests.

### Perceived risk and trust

Extensions of the TAM include perceived risk and trust as additional predictors of intentions to use a new technology.^26^ Perceived risk for health technology has been defined as the “degree of uncertainty related to use of the medium that is beyond the control of the information manager associated with the eHealth service.”^19^ Women may perceive OFCs as risky, as past research found that individuals who use telemedicine services for sexual health and medical abortions express concerns about privacy, confidentiality, and the risks associated with discussing and storing their personal health information online.^19,33^ Women may also perceive using reproductive hormone SCTs as risky, as past work has found that patients express concerns about privacy with self-collection genetic tests.^34^

Trust in health technology has been defined as “the belief that the other party will behave responsibly and will not attempt to exploit the vulnerabilities of the user,”^19^ and a meta-analysis on the addition of trust to the TAM found strong relationships between trust and the major TAM variables (perceived ease of use, perceived usefulness, and behavioral intentions), suggesting that trust is important in the utilization of new technologies.^35^ Furthermore, trust improves an individual's beliefs about that online entity, which in turn attenuates perceptions of risk associated with using that online entity; thus, trust is posited to reduce perceptions of risk.^25^ Trust also likely plays a large role in use of reproductive health services, as reproductive health and infertility are fraught with stigma.^12,36^ Individuals may delay or avoid treatment to hide stigmatizing information from others,^36,37^ underscoring the importance of confidentiality for OFCs and reproductive hormone SCTs. We are not aware of any extant research on levels of risk and trust in these new reproductive health technologies. Using this extension of the TAM, we hypothesized the following:

H4: Perceived risk is negatively related to intentions to use (a) online fertility consultations and (b) reproductive hormone self-collection tests.

H5: Perceived trust is positively related to usefulness of (a) online fertility consultations and (b) reproductive hormone self-collection tests.

H6: Perceived trust is positively related to ease of use of (a) online fertility consultations and (b) reproductive hormone self-collection tests.

H7: Perceived trust is negatively related to perceived risk of (a) online fertility consultations and (b) reproductive hormone self-collection tests.

H8: Perceived trust is positively related to intentions to use (a) online fertility consultations and (b) reproductive hormone self-collection tests.

### Subjective norms

Past work has shown that the TAM is a more useful model for predicting the acceptance of a new technology^25,38,39^ than the theory of reasoned action (TRA)^40^ and the theory of planned behavior (TPB)^39^; however, integration of subjective norms from the TRA/TPB may be an important addition to the TAM (see the TAM 2^41^) and is supported by meta-analytic data.^27^ Subjective norms include an individual's beliefs about whether or not people important to them think they should perform the behavior. Women's use of fertility health care and, subsequently, new reproductive health technologies may be influenced by other people who are important to them, as reproductive decision-making often involves romantic partners^42^ and other family members.^43,44^ In line with this extension of the TAM, we thus hypothesized the following:

H9: Subjective norms are positively related to intentions to use (a) online fertility consultations and (b) reproductive hormone self-collection tests.

### Responsibility

Perceptions of responsibility may be a key predictor of intentions to engage in health-related behaviors.^28^ Theoretical frameworks, including the Norm Activation Model,^45^ highlight the role of responsibility in predicting behavior. Likewise, meta-analyses have found that the inclusion of personal responsibility in the TPB explains an additional 3%–4% of the variation in intentions.^46,47^ Empirical work also suggests that perceptions of responsibility influence health-related behaviors, including obtaining a mammogram.^48^ To date, we are unaware of any research that has examined women's perceptions of responsibility to use OFCs and reproductive hormone SCTs or the role of responsibility in predicting technology acceptance. We thus propose an extension of the TAM to include responsibility with the following hypothesis:

H10: Responsibility is positively related to intentions to use (a) online fertility consultations and (b) reproductive hormone self-collection tests.

## Materials and Methods

Participants were recruited through an electronic newsletter that was distributed through a national women's magazine in March 2019.^†^. Women who have voluntarily added themselves to this national women's magazine's email list received the recruitment message and survey link. No incentive was provided for participation. Eligible participants were 18–59 years of age, identified as women, and lived in the United States. After providing informed consent, an online questionnaire assessed participants' perceptions of usefulness, ease of use, trust, risk, subjective norms, personal responsibility to learn about fertility, and intentions to use for both OFCs and reproductive hormone SCTs. The study was approved by Western IRB, a third-party institutional review board accredited by the Association for the Accreditation of Human Research Protection Programs.

### Measures

All variables were measured using 5-point Likert-type items. Each variable was measured for both OFCs and reproductive hormone SCTs. Participants were asked to indicate the extent to which they agreed (“1” = “Strongly disagree” to “5” = “Strongly agree”) with each statement. Scale descriptives are displayed in Table 2.

#### Perceived usefulness

Perceived usefulness was measured by two items^19^: (1) I think a/n (online consultation with a fertility specialist/home fertility test) would be useful and (2) a/n (online consultation with a fertility specialist/home fertility test) would be beneficial.

#### Perceived ease of use

Perceived ease of use was measured by two items^19^: (1) I think a/n (online consultation with a fertility specialist/home fertility test) would be easy to use and (2) it would be easy for me to learn how to use a/n (online consultation with a fertility specialist/home fertility test).

#### Perceived risk

Perceived risk for was measured by two items^26^: (1) I am concerned about using a/n (online consultation with a fertility specialist/home fertility test) because storing my health information online is risky and (2) I am concerned about using a/n (online consultation with a fertility specialist/home fertility test) because storing my health information online is a threat to my privacy.

#### Perceived trust

Perceived trust was measured by two items^26^: (1) I trust that a/n (online consultation with a fertility specialist/home fertility test) would keep my health information secure and (2) I am confident that I could trust in a/n (online consultation with a fertility specialist/home fertility test).

#### Subjective norms

Subjective norms were measured by two items^49^: (1) people who are important to me would think that I should use a/n (online consultation with a fertility specialist/home fertility test) and (2) people whose opinions I value would think that I should use a/n (online consultation with a fertility specialist/home fertility test).

#### Responsibility

Perceived responsibility to learn more about fertility was measured by two items^28^: (1) I feel a personal responsibility to learn more about my hormones with a/n (online consultation with a fertility specialist/home fertility test) and (2) learning more about my hormones with a/n (online consultation with a fertility specialist/home fertility test) is my responsibility.

#### Intentions

Intentions to use were measured by two items^50^: (1) I would buy a/n (online consultation with a fertility specialist/home fertility test) and (2) I intend to buy a/n (online consultation with a fertility specialist/home fertility test).

### Data analysis

To evaluate our hypotheses, we tested the theoretical models for OFCs and reproductive hormone SCTs (visualized in Fig. 1) by first analyzing both full measurement models. We then used a hybrid approach to test both structural models.^51^ We included one absolute fit statistic to test model fit, *χ*^2^,^51,52^ and additional goodness–of-fit tests, including the comparative fit index (CFI)^51,52^ and the root mean squared error (RMSEA).^53^ AMOS 22.0 with maximum likelihood estimation was used for all measurement and structural analyses; *p* ≤ 0.05 was set as the *a priori* significance level for hypothesis testing.

## Results

**FIG. 2.**
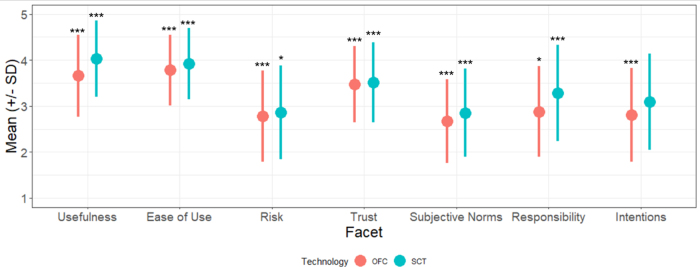
Women's perceptions of online fertility consultations and reproductive hormone SCTs. Note: Asterisks indicate a statistically significant difference (*p < 0.05, ***p < 0.001) from the scale midpoint.

A total of 327 women completed the survey. Participants ranged in age from 18 to 59 (mean = 34.11, standard deviation [SD] = 6.64). Participant demographics are displayed in Table 1. The majority of women had completed a 4-year degree or graduate school (81.7%) and self-identified as white (75.8%) and heterosexual (95.1%). One-third (33.33%) of participants were previously diagnosed with or reported meeting diagnostic criteria for infertility. Means, standard deviations (SDs), and bivariate correlations between the theoretical variables are displayed in Table 2 and visualized in Figure 2. A series of one-sample *t*-tests were used to analyze the means of each scale against the midpoint (3) and revealed that women had high perceptions of usefulness, ease of use, and trust and low perceptions of risk and subjective norms for both OFCs and reproductive hormone SCTs. Women indicated low perceptions of responsibility to use OFCs, but high responsibility to use reproductive hormone SCTs. Women reported low intentions to use OFCs; intentions to use reproductive hormone SCTs did not differ from the midpoint.

**Table 1.** Participant Demographics

	n	%
Education		
Less than high school	1	0.30
High school graduate	6	1.83
Some college	39	11.92
2-year degree	14	4.28
4-year degree	136	41.59
Attended and/or completed graduate school	131	40.06
Racial/ethnic background (note: could select more than one)		
American Indian or Alaskan	2	0.61
Asian	18	5.50
Black or African American	28	8.56
Native Hawaiian or other Pacific Islander	3	0.92
White	248	75.84
Hispanic or Latino	44	13.46
Other	6	1.83
Prefer not to say	3	0.92
Sexual orientation		
Heterosexual or straight	311	95.11
Gay or lesbian	1	0.30
Bisexual	15	4.58
Relationship status		
Single (never married)	55	16.82
In a monogamous, dating relationship	45	13.76
In an open, dating relationship	1	0.30
In a domestic partnership or living with a partner	35	10.70
Married	184	56.27
Widowed	0	0
Divorced	6	1.83
Separated	1	0.30
Geographic region		
Northeast	62	18.96
Midwest	60	18.34
South	101	30.89
West	97	29.66
Pacific	0	0
Did not indicate	5	1.53
Fertility status		
Fertile	218	66.66
Infertile	109	33.33

**Table 2.** Means, Standard Deviations, and Bivariate Correlations of Theoretical Variables

	1	2	3	4	5	6	7	Mean	SD	α
OFC usefulness	—							3.66	0.89	0.96
OFC ease of use	0.65^*^	—						3.78	0.77	0.86
OFC risks	−0.19^*^	−0.21^*^	—					2.78	0.99	0.94
OFC trust	0.60^*^	0.60^*^	−0.49^*^	—				3.47	0.83	0.85
OFC subjective norms	0.46^*^	0.33^*^	−0.16^*^	0.47^*^	—			2.67	0.91	0.97
OFC responsibility	0.50^*^	0.41^*^	−0.10^*^	0.47^*^	0.65^*^	—		2.88	0.99	0.86
OFC intentions	0.58^*^	0.44^*^	−0.22^*^	0.49^*^	0.59^*^	0.60^*^	—	2.81	1.02	0.80
SCT usefulness	—							4.03	0.83	0.93
SCT ease of use	0.45^*^	—						3.92	0.77	0.80
SCT risks	−0.22^*^	−0.19^*^	—					2.86	1.02	0.89
SCT trust	0.59^*^	0.55^*^	−0.36^*^	—				3.51	0.87	0.80
SCT subjective norms	0.35^*^	0.30^*^	−0.12^*^	0.34^*^	—			2.85	0.96	0.96
SCT responsibility	0.51^*^	0.34^*^	−0.12^*^	0.46^*^	0.54^*^	—		3.28	1.05	0.82
SCT intentions	0.58^*^	0.43^*^	−0.16^*^	0.46^*^	0.53^*^	0.67^*^	—	3.09	1.05	0.84

^*^ *p* < 0.05.

OFC, online fertility consultation; SCT, self-collection test; SD, standard deviation.

**FIG. 3.**
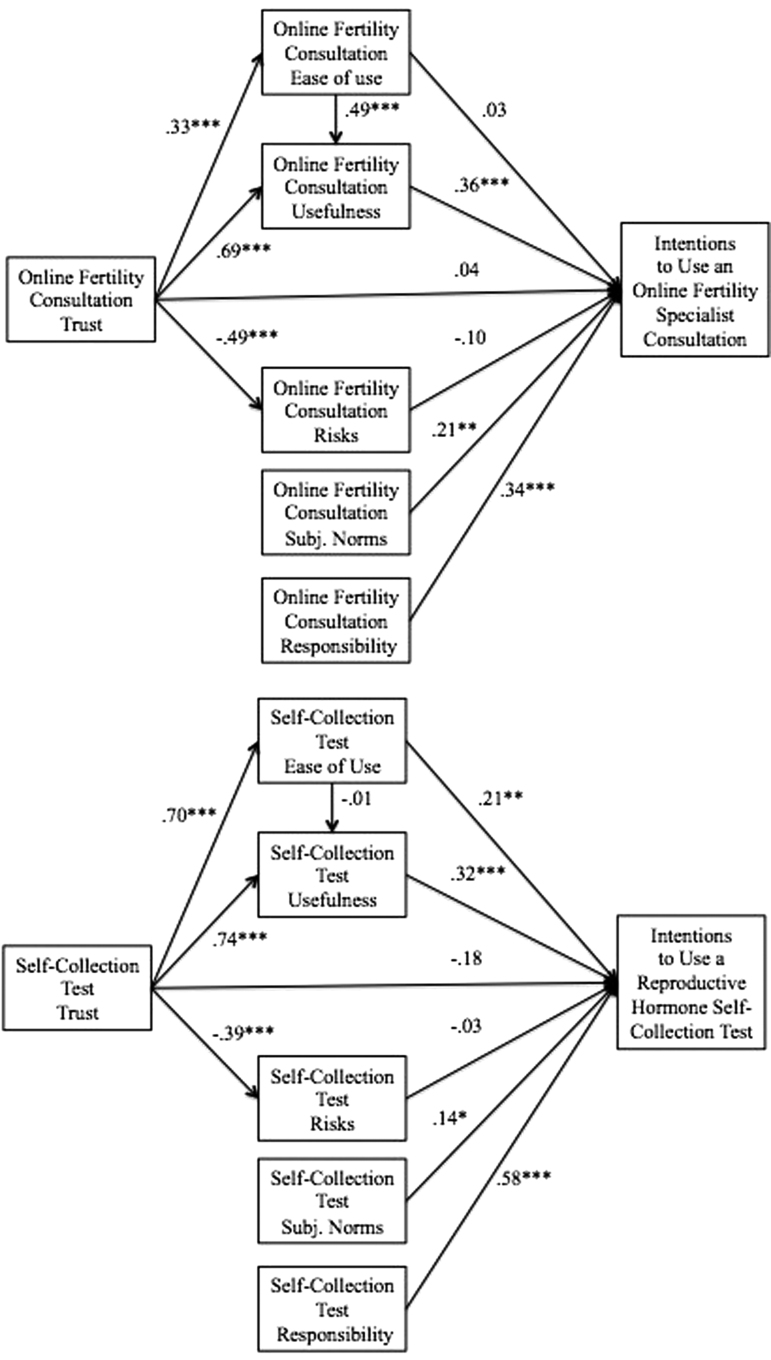
Structural equation modeling results predicting intentions to use an online fertility specialist consultation and reproductive hormone SCT. *p < 0.05, **p < 0.01, ***p < 0.001.

The results of the structural equation modeling for OFCs are depicted in Figure 3 and Table 3. The power for testing the measurement model was excellent (0.99; with alpha = 0.05, *df* = 56, *n* = 327, null RMSEA = 0.05, and alt RMSEA = 0.08).^54^ The measurement model had adequate model fit (*χ*^2^ (56, *n* = 327) = 175.18, *p* < 0.001; CFI = 0.97; RMSEA = 0.081, 90% confidence interval (CI) (0.067, 0.094), *p*-close <0.01). The power for testing the structural model was excellent (0.99; with alpha = 0.05, *df* = 64, *n* = 327, null RMSEA = 0.05, and alt RMSEA = 0.08).^54^ The structural model had adequate model fit (*χ*^2^ (64, *n* = 327) = 234.86, *p* < 0.001; CFI = 0.96; RMSEA = 0.090, 90% CI (0.078, 0.103), *p*-close <0.01). Seven of the 10 hypothesized direct paths were statistically significant.

**Table 3.** Structural Equation Model Results

	β	B	SE
OFCs			
H1a, OFCs usefulness → intentions to use	0.36^***^	0.31	0.06
H2a, OFCs ease of use → intentions to use	0.03	0.03	0.08
H3a, OFCs ease of use → usefulness	0.49^***^	0.59	0.08
H4a, OFCs risk → intentions to use	−0.10	−0.07	0.04
H5a, OFCs trust → usefulness	0.33^***^	0.38	0.08
H6a, OFCs trust → ease of use	0.69^***^	0.65	0.05
H7a, OFCs trust → risk	−0.49^***^	−0.65	0.07
H8a, OFCs trust → intentions to use	0.04	0.04	0.09
H9a, OFCs subj. norms → intentions to use	0.21^**^	0.18	0.06
H10a, OFCs responsibility → intentions to use	0.34^***^	0.29	0.06
Reproductive hormone SCTs			
H1b, SCTs usefulness → intentions to use	0.32^***^	0.34	0.08
H2b, SCTs ease of use → intentions to use	0.21^**^	0.30	0.11
H3b, SCTs ease of use → usefulness	−0.01	−0.02	0.12
H4b, SCTs risk → intentions to use	−0.03	−0.03	0.04
H5b, SCTs trust → usefulness	0.74^***^	0.80	0.10
H6b, SCTs trust → ease of use	0.70^***^	0.56	0.06
H7b, SCTs trust → risk	−0.39^***^	−0.53	0.08
H8b, SCTs trust → intentions to use	−0.18	−0.21	0.14
H9b, SCTs subj. norms → intentions to use	0.14^*^	0.13	0.05
H10b, SCTs responsibility → intentions to use	0.58^***^	0.62	0.08

^*^ *p* < .05, ^**^p < .01, ^***^p < .001.

SE, standard error.

The results of the structural equation modeling for reproductive hormone SCTs are depicted in Figure 3 and Table 3. The power for testing the measurement model was excellent (0.99; with alpha = 0.05, *df* = 56, *n* = 327, null RMSEA = 0.05, and alt RMSEA = 0.08).^54^ The measurement model has good model fit (*χ*^2^ (56, *n* = 327) = 114.91, *p* < 0.001; CFI = 0.98; RMSEA = 0.057, 90% CI (0.042, 0.072), *p*-close = 0.21). The power for testing the structural model was excellent (0.99; with alpha = 0.05, *df* = 64, *n* = 327, null RMSEA = 0.05, and alt RMSEA = 0.08).^54^ The structural model had good model fit (*χ*^2^ (64, *n* = 327) = 140.88, *p* < 0.001; CFI = 0.98; RMSEA = 0.061, 90% CI (0.047, 0.074), *p*-close = 0.09). Seven of the 10 hypothesized direct paths were statistically significant.

In summary, women's intentions to use OFCs were significantly predicted by perceptions of responsibility to learn about their fertility with OFCs, beliefs that other people important to them think they ought to use OFCs, and perceptions that OFCs are useful. Women's intentions to use reproductive hormone SCTs were significantly predicted by perceptions of responsibility to learn about their fertility with reproductive hormone SCTs, beliefs that other people important to them think they ought to use reproductive hormone SCTs, and perceptions that reproductive hormone SCTs are useful and easy to use.

## Discussion

Women have historically faced many difficulties accessing reproductive health care services, including fertility specialists and reproductive hormone testing.^8,9,16,17^ The coronavirus disease 2019 (COVID-19) pandemic will also likely exacerbate women's access to sexual and reproductive care.^55^ OFCs and reproductive hormone SCTs^4^ can help overcome these barriers and enable women to gain critical fertility information. The goal of this article was therefore to examine women's perceptions of OFCs and reproductive hormone SCTs and factors that influence women's intentions to use these new reproductive health technologies. Our results support our overall extension of the TAM to include perceived risk, trust, subjective norms, and responsibility.

Perceptions of responsibility were the largest predictor of intentions to use both OFCs and reproductive hormone SCTs. These findings suggest that perceptions of responsibility play an important role in the acceptance of new reproductive health technologies and thus support our theoretical extension of the TAM. Practically speaking, these results suggest that women would be more likely to utilize OFCs and reproductive hormone SCTs, and thus gain the benefits from doing so through heightened perceptions that learning about their fertility is their responsibility. Importantly, however, women have historically been burdened with a disproportionate amount of responsibility for reproductive health care,^56^ and past work has found that perceptions of responsibility for health can unintentionally lead to guilt, shame, or frustration when an individual is not able to adopt a recommended practice.^57^ Furthermore, responsibility may be central to the formation of stigma beliefs,^58^ and reproductive health^36^ and infertility^12^ are already stigmatized. Together, this suggests that caution should be taken when considering increasing perceptions of responsibility to motivate the acceptance and use of OFCs and reproductive hormone SCTs. Perceptions of subjective norms and usefulness, which were also significant predictors of intentions to use both reproductive health technologies, should thus be considered.

Subjective norms predicted intention to use both OFCs and reproductive hormone SCTs, indicating that women's beliefs that other people who are important to them think they ought to use these new reproductive health technologies play an important role in women's acceptance and intentions to do so. These results support previous extensions of the TAM that include subjective norms.^27,41^ These results also suggest that reproductive decisions are not solely a function of women alone, but instead include important others such as romantic partners, family members, or health care providers, which aligns with previous work.^42^ It is possible to increase these beliefs by encouraging women who have already used these technologies to share their experiences with their friends. For example, women who received a medical abortion through telemedicine were more likely to recommend the service to a friend than those who saw a physician in person.^30^ Women may be further motivated to use these new reproductive health technologies if health care providers also suggest they do.

Perceptions of usefulness also predicted intentions to use both OFCs and reproductive hormone SCTs, which indicates that women's intentions to use these new reproductive technologies are related to their belief that doing so is beneficial. Past research suggests that both online consultations^22,23^ and reproductive hormone SCTs^4^ are as useful as traditional in-person consultations and venipuncture sampling. Our results suggest that women do recognize these benefits, as they perceived both OFCs and reproductive hormone SCTs as useful. Health care providers or public health campaigns could increase the use of OFCs and reproductive hormone SCTs by emphasizing that, in this era of delayed childbirth,^1^ these reproductive health technologies may facilitate informed decisions that might aid women in achieving their reproductive goals.

Ease of use was a significant predictor of intentions to use reproductive hormone SCTs, but not intentions to use OFCs. Both video-chat applications and online health care consultations have been steadily increasing.^59^ Thus, the ease with which many people engage in these technologies may modulate the influence of perceptions of ease of use on the acceptance of OFCs. SCTs, however, are less common as, to date, only four companies offer this testing.^60,61^ Most reproductive hormone SCTs require a fingerstick method to collect a blood sample; thus, women's intentions to use this technology is justifiably influenced by their perceptions of their ability to do so.

Interestingly, our study found that perceptions of risk and trust do not predict intentions to use OFCs or reproductive hormone SCTs, and, overall, perceptions of risk were low and perceptions of trust were high for both technologies. These results are surprising, as previous research notes that patients have concerns about privacy and risks associated with storing their personal health information online with telemedicine services^19,33^ and with SCTs.^34^ Likewise, the stigma in reproductive health^12,36^ underscores the need for patients to trust in fertility health care services. However, past work has also noted that online consultations may be more readily accepted if patients are provided with clear and accessible privacy policies.^33^ Furthermore, with the increasing use of the Internet for these services,^59^ this information may already be more readily available, and patients may be more accustomed to using technology in this way.^59^

Importantly, OFCs and reproductive hormone SCTs may be most beneficial in promoting positive reproductive health and fertility outcomes when used together. Indeed, OFCs may be particularly beneficial as a way to help women understand their results from reproductive hormone SCTs^62^ or as psychological counseling sessions after unexpected or undesired results. For example, a recent small-scale ethnographic study found that their participants believed the reproductive hormone SCTs could be empowering; however, some participants were uncertain about how to interpret the test results and the appropriate next steps to take.^29^ Pairing a reproductive hormone SCT with an online fertility specialist consultation could thus improve patient experience and ability to use the reproductive hormone SCT results effectively.

Finally, as mentioned, the COVID-19 pandemic may result in additional barriers for women to access reproductive health care services.^55^ For example, reproductive health providers and clinics may be deemed “nonessential” and redirected to respond to COVID-19.^63^ Thus, telemedicine services may be ideal for limiting the number of patients within a given hospital or clinic and preventing unnecessary human exposures, while still promoting high-quality health care.^64^ Together, this underscores the increasing importance of providing online reproductive health services, including OFCs and reproductive hormone-SCTs. Promoting intentions to use these online services has therefore become increasingly important and relevant.

### Limitations and future directions

This is the first study that directly examines women's perceptions of OFCs and reproductive hormone SCTs and applies the TAM and its extensions to examine women's intentions to utilize these new reproductive health technologies. However, this sample was homogenous with respect to age, education, sexual orientation, and race. Prior work has shown that demographic factors, particularly age,^65^ may modulate telemedicine utilization; as such, future research should examine perceptions of fertility-related telemedicine services in more diverse samples. We were not able to test the effect of certain factors related to reproductive health status, like current pregnancy status, on perceptions of fertility-related telemedicine services. Thus, future work should make sure to examine such factors. We also analyzed predictors of women's intentions to use OFCs and hormone SCTs, rather than predictors of women's actual use of these technologies. As these services become increasingly available and widespread in use, future work should examine women's actual utilization of OFCs and reproductive hormone SCTs. Finally, we used two items to measure each theoretical construct in our extension of the TAM. The measurement models for both OFCs and reproductive hormone SCTs had adequate model fit; however, future work should measure each variable with additional items.

## Conclusions

As age of first childbirth is delayed^1^ and total fertility rates decrease,^2^ it is particularly important to provide women with access to reproductive health care services that facilitate their ability to make informed reproductive decisions. Online consultations with fertility specialists and reproductive hormone SCTs present new and unique opportunities to overcome the barriers women have historically faced accessing these services. To promote the use of these new reproductive health technologies, health campaigns should originate from important others (such as physicians) and should emphasize that these technologies are useful and easy to use. It is our hope that widespread utilization of these reproductive health technologies can aid in reducing the number of women who suffer from infertility by increasing awareness and treatment.

## Abbreviations Used

CFI: comparative fit index
CI: confidence interval
COVID-19: coronavirus disease 2019
OFC: online fertility consultation
RMSEA: root mean squared error
SCT: self-collection test
SD: standard deviation
SE: standard error
TAM: technology acceptance model
TPB: theory of planned behavior
TRA: theory of reasoned action

## Notes

^†^ This magazine is not related to this study's funding source.
